# Using Robson's Ten‐Group Classification System for comparing caesarean section rates in Europe: an analysis of routine data from the Euro‐Peristat study

**DOI:** 10.1111/1471-0528.16634

**Published:** 2021-02-01

**Authors:** J Zeitlin, M Durox, A Macfarlane, S Alexander, G Heller, M Loghi, J Nijhuis, H Sól Ólafsdóttir, E Mierzejewska, M Gissler, B Blondel, the Euro‐Peristat Network, Gerald Haidinger, Jeannette Klimont, Gisèle Vandervelpen, Wei‐Hong Zhang, Evelin Jordanova, Rumyana Kolarova, Boris Filipovic‐Grcic, Zeljka Drausnik, Urelija Rodin, Theopisti Kyprianou, Vasos Scoutellas, Petr Velebil, Laust Mortensen, Luule Sakkeus, Anna Heino, Anne Chantry, Catherine Deneux Tharaux, Nicholas Lack, Aris Antsaklis, István Berbik, Sheelagh Bonham, Karen Kearns, Izabela Sikora, Marina Cuttini, Janis Misins, Irisa Zile, Jelena Isakova, Audrey Billy, Sophie Couffignal, Aline Lecomte, Guy Weber, Miriam Gatt, Peter Achterberg, Lisa Broeders, Ashna Hindori‐Mohangoo, Rupali Akerkar, Kari Klungsøyr, Katarzyna Szamotulska, Henrique Barros, Mihai Horga, Vlad Tica, Jan Cap, Natasa Tul, Ivan Verdenik, Francisco Bolumar, Mireia Jané, Adela Recio Alcaide, Maria José Vidal, Oscar Zurriaga, Karin Källén, Anastasia Nyman, Sylvan Berrut, Mélanie Riggenbach, Tonia A Rihs, Lucy Smith, Rachael Wood, Marie Delnord, Alice Hocquette

**Affiliations:** ^1^ CRESS, Obstetrical Perinatal and Paediatric Epidemiology Research Team, EPOPe, INSERM, INRA Universite de Paris Paris France; ^2^ Centre for Maternal and Child Health Research School of Health Sciences City, University of London London UK; ^3^ Perinatal Epidemiology and Reproductive Health Unit CR2, School of Public Health ULB Brussels Belgium; ^4^ Institute for Quality Assurance and Transparency in Health Care Berlin Germany; ^5^ Directorate for Social Statistics and Welfare Italian Statistical Institute (ISTAT) Rome Italy; ^6^ Department of Obstetrics & Gynaecology Maastricht University Medical Centre MUMC+ Maastricht The Netherlands; ^7^ Department of Obstetrics and Gynaecology Landspitali University Hospital Reykjavik Iceland; ^8^ Department of Epidemiology and Biostatistics National Research Institute of Mother and Child Warsaw Poland; ^9^ THL Finnish Institute for Health and Welfare Helsinki Finland; ^10^ Karolinska Institute Stockholm Sweden

**Keywords:** Caesarean birth, Europe, health information systems, perinatal health indicators, Robson classification, Ten‐Group Classification System

## Abstract

**Objective:**

Robson's Ten Group Classification System (TGCS) creates clinically relevant sub‐groups for monitoring caesarean birth rates. This study assesses whether this classification can be derived from routine data in Europe and uses it to analyse national caesarean rates.

**Design:**

Observational study using routine data.

**Setting:**

Twenty‐seven EU member states plus Iceland, Norway, Switzerland and the UK.

**Population:**

All births at ≥22 weeks of gestational age in 2015.

**Methods:**

National statistical offices and medical birth registers derived numbers of caesarean births in TGCS groups.

**Main outcome measures:**

Overall caesarean rate, prevalence and caesarean rates in each of the TGCS groups.

**Results:**

Of 31 countries, 18 were able to provide data on the TGCS groups, with UK data available only from Northern Ireland. Caesarean birth rates ranged from 16.1 to 56.9%. Countries providing TGCS data had lower caesarean rates than countries without data (25.8% versus 32.9%, *P* = 0.04). Countries with higher caesarean rates tended to have higher rates in all TGCS groups. Substantial heterogeneity was observed, however, especially for groups 5 (previous caesarean section), 6, 7 (nulliparous/multiparous breech) and 10 (singleton cephalic preterm). The differences in percentages of abnormal lies, group 9, illustrate potential misclassification arising from unstandardised definitions.

**Conclusions:**

Although further validation of data quality is needed, using TGCS in Europe provides valuable comparator and baseline data for benchmarking and surveillance. Higher caesarean rates in countries unable to construct the TGCS suggest that effective routine information systems may be an indicator of a country's investment in implementing evidence‐based caesarean policies.

**Tweetable abstract:**

Many European countries can provide Robson's Ten‐Group Classification to improve caesarean rate comparisons.

## Tweetable abstract

Many European countries can provide Robson's Ten‐Group Classification to improve caesarean rate comparisons.

## Introduction

Caesarean birth rates differ by a factor of three in European countries from just over 15% to over 45%, as shown in recent data from the Euro‐Peristat project.^1^ Many reasons have been suggested for this, including differences in the characteristics of childbearing women, such as maternal age or the prevalence of comorbidities, clinicians' interpretation of evidence about the management of risks during pregnancy, women's preferences or socio‐cultural norms about mode of delivery, fear of litigation and the organisation and financing of maternity care.^2^, ^3^, ^4^ Benchmarking between countries has the potential to yield valuable insights into these underlying causes as well as the consequences for the health of mothers and babies.

As the overall caesarean rate conflates multiple groups with differing levels of risk, subdividing it by risk group is an important first step for comparative analyses. The Ten‐Group Classification System (TGCS), proposed in 2001 by Michael Robson, provides a clinically relevant framework for assessing differences in the caesarean rate and, as stated by Robson, ‘serves as the initial structure within which additional epidemiological variables, processes, perinatal events and outcomes in addition to caesarean sections can be analysed.’^5^, ^6^ It has been recommended by the World Health Organization (WHO) for comparisons of the caesarean rate between hospitals^7^ and is increasingly used for comparisons of rates between healthcare units and countries and trends over time.^8^, ^9^, ^10^, ^11^, ^12^ The classification divides women into ten mutually exclusive groups, two of which can be disaggregated further into an expanded 12‐group version. These groups cover all situations based on six maternal and fetal characteristics (parity, gestational age, plurality, fetal presentation, mode of onset and previous caesarean section).^13^

The Euro‐Peristat project, which aims to monitor perinatal health indicators in Europe, recommends the presentation of the caesarean rate by selected risk groups, which include those in the TGCS, but up to now, these have been collected and presented separately.^14^ In this study, we aimed to assess whether the data in routine systems in Europe could be used to construct the TGCS and the contribution of this framework to understanding differences between countries in their caesarean rates.

## Methods

Data come from the most recent collection of data, on births in 2015, by the Euro‐Peristat project.^1^, ^15^ Euro‐Peristat developed a set of 30 indicators on perinatal health based on national‐level data, which have been used to produce three European perinatal health reports, including a report on core indicators in 2015.^15^ Using a common protocol, data are compiled from routine sources such as medical birth registers, civil registration of births, hospital discharge systems and nationally representative survey data.^1^, ^16^ All 27 current EU member states, as well as Iceland, Norway, Switzerland and the UK, participated. UK data were submitted from constituent countries so England, Wales, Northern Ireland and Scotland are presented separately, making 34 countries in total. In each country, one scientific committee member has overall responsibility for coordinating data collection, but in most countries, there is a team including several data providers.

Euro‐Peristat requests data for all stillbirths and live births at 22 or more weeks of gestation, or weighing 500 g or more if gestational age is missing. When countries cannot provide data using this recommended inclusion threshold, they are asked to provide data using their national inclusion criteria along with a description of these criteria. Most provided data using the 22‐week threshold for live births, but other thresholds exist for stillbirths in some countries, such as a 500‐g limit or a 24‐week threshold.^17^ Some countries, which did not have data for 2015, provided data for other years: the French data come from a national survey based on a representative sample of births in 2016 and the Swiss data are for 2014. Swedish data in the Euro‐Peristat report were for 2014, but were updated to 2015 for this study.

Mode of delivery is one of Euro‐Peristat's core indicators and is compiled separately by risk sub‐groups based on parity, plurality, fetal presentation, previous caesarean section and gestational age.^14^ Caesarean birth rates are calculated as a proportion of all stillbirths and live births, following the convention of international organisations, such as WHO and the Organisation for Economic Co‐operation and Development. For the data collection exercise based on 2015 births, data about the TGCS were compiled using a pretested, standardised data collection table. The ten groups, including sub‐groups are shown in Box 1.

Box 1 **Robson's Ten‐Group Classification System (TGCS), including sub‐groups**

(1) Nulliparous, singleton, cephalic, term (≥37^+0^ weeks) births in spontaneous labour;

(2) Nulliparous, singleton, cephalic, term births with (2a) induced labour or (2b) prelabour caesarean section;

(3) Multiparous, singleton, cephalic, term births without previous caesarean section in spontaneous labour;

(4) Multiparous, singleton, cephalic, term births without previous caesarean section with (4a) induced labour or (4b) prelabour caesarean section;

(5) Previous caesarean section, singleton, cephalic, term births;

(6) Nulliparous singleton breech births;

(7) Multiparous singleton breech births, including previous caesarean section;

(8) Multiple pregnancies, including previous caesarean section;

(9) Transverse and oblique lies, including previous caesarean section;

(10) Preterm (<37^+0^ weeks), singleton, cephalic births, including previous caesarean section.

Data providers filled in the numbers of total births and the numbers of caesarean sections in each group separately. Some providers used an experimental protocol whereby data were provided on the necessary items in disaggregated tables and a customised STATA programme^18^ was used to produce the aggregated table. Because data on caesarean sections are compiled by babies born rather than by women giving birth, we retained this convention for the TGCS. The category of multiple births, Group 8, was therefore divided by two to estimate the number of women. Some misclassification therefore exists in this group because of multiple births with discordant modes of delivery as well as triplets and higher‐order multiples. The other nine categories are not affected by whether data are collected by births or women.

Euro‐Peristat definitions were applied to the TGCS. Labour induction is defined as initiation of uterine contractions by medical or surgical means before the onset of labour. Prelabour caesarean sections are those occurring before the onset of labour, but as some countries use a classification based on whether caesarean sections are elective or emergency, Euro‐Peristat combines prelabour with elective caesarean sections and caesarean sections during labour with emergency caesarean sections.^14^

All data were cross‐checked for potential discrepancies with data on total numbers of births, total number of caesarean sections, multiple births, preterm births, parity compiled for other indicators and errors were corrected by the data providers. The final data tables were verified by each of the country teams. Data were then formatted into the recommended tables for presenting the TGCS for each country.^19^

### Missing data

The percentages of missing data were calculated and reported for each country. Countries with high proportions of missing data indicative of incomplete reporting of some Robson categories were not included in the study. For other countries, we imputed the numbers of missing cases using the observed distributions to allow comparison of the size of the groups and estimate their contribution to the overall caesarean rate. We first imputed the missing categories among caesarean births and then imputed missing observations for non‐caesarean births (details for each country in Supplementary material, Tables S1–S18).

### Analysis strategy

We described the overall caesarean birth rate for each country as calculated by Euro‐Peristat in its reports and assessed countries' capacity to provide data using the TGCS groups. We then compared caesarean birth rates by ability to provide data on TGCS using an independent sample two‐sided *t*‐test. Greece does not have national data on the caesarean rate, so its rate was estimated from a WHO working group report (www.euro.who.int/en/countries/greece/news/news/2016/11/greece‐commits‐to‐addressing‐excessive‐reliance‐on‐caesarean‐sections).^20^

To compare the groups between countries, we produced graphs for each dimension of the recommended tables: the size of each group as a percentage of the total population of women having a live birth or stillbirth, the caesarean rate in each group and the absolute contribution of each group to the overall caesarean rate. To simplify the interpretation of this information, we ordered countries by their overall caesarean section rate from low to high. Because of the large numbers of countries and categories, it was not considered appropriate to use significance tests for comparisons between groups and countries. However, we drew attention to countries where denominators fell below 100 women for some groups (Cyprus, Estonia, Iceland, Luxembourg, Malta, Northern Ireland and Slovenia). Spearman rank correlations were used to show associations between the TGCS groups.

### Ethics permissions

Euro‐Peristat received authorisations for its 2015 core database from the French Advisory Committee on Use of Health Data in Medical Research (N°17‐048, 30 March 2017) and the French National Commission for Data Protection and Liberties (DR‐2019‐089, 26 March 2019).

### Patient participation

This analysis did not involve patients or patient groups.

### Core outcome sets

The TGCS is part of core outcome sets.^21^

### Funding

Euro‐Peristat receives support as part of the InfAct Joint Action (Grant no. 801553) and data collection was partially funded as part of the BridgeHealth Project (Grant no. 665691), Public Health Programme, Consumers, Health, Agriculture and Food Executive Agency (CHAFEA). The funding agency was not involved in analysis or interpretation of results.

## Results

Caesarean rates in 2015, based on total births, ranged from 16.1% (Iceland) to 56.9% (Cyprus) as shown in Table 1. Seventeen out of 31 countries (55%) provided national data for the TGCS groups. In the UK, many data were missing in all constituent countries except Northern Ireland, which was included separately, bringing the number of countries with full data available for the analysis to 18. Austria could provide all items except for previous caesarean section, so data were missing for groups 3 through 5 (>45% of women), as a result Austria was excluded from the analysis. In most other countries with data, percentages of missing data were low, less than 2%, although these were slightly higher in Switzerland (5%), Italy (3%) and the Netherlands (3%). The countries that could provide data for the TGCS had lower caesarean rates, on average, than countries without these data (25.8% versus 32.9%, *P* = 0.04).

**Table 1:** Caesarean birth rates in European countries and availability of data for the Ten‐Group Classification System in 2015

Country	Total births	Caesarean birth rate (% of live births)^a^	Data for Ten‐Group Classification System	Missing data
Austria	83 884	29.7	Partial^b^	(46.6%)^d^
Belgium	122 838	21.3	Yes	1.8%
Bulgaria (2014)	68 079	43.0	No	–
Croatia	37 428	21.6	No	–
Cyprus	9425	56.9	Yes	1.5%
Czech Republic	111 162	26.1	No	–
Denmark	57 847	21.6	Yes	0.9%
Estonia	13 961	19.5	Yes	0.01%
Finland	55 759	16.4	Yes	0.1%
France (Survey, 2016)	13 311	20.2	Yes	0.5%
Germany	728 496	32.2	Yes	0.02%
Greece	92 159	≈50.0^d^	No	–
Hungary	92 206	39.0	No	–
Iceland	4098	16.1	Yes	1.8%
Ireland	65 913	31.3	No	–
Italy	486 557	35.4	Yes	3.2%
Latvia	21 826	22.0	Yes	0.0%
Lithuania	31 601	21.9	No^e^	–
Luxembourg	6862	32.7	Yes	0.04%
Malta	4453	32.0	Yes	0.0%
Netherlands	169 234	17.4	Yes	3.0%
Norway	59 928	16.5	Yes	0.4%
Poland (2014)	369 709	42.2	No	–
Portugal	86 048	32.9	No	–
Romania	201 760	46.9	No	–
Slovakia	55 824	31.1	No	–
Slovenia	20 336	21.2	Yes	0.2%
Spain	421 590	24.6	No	–
Sweden^f^	116 667	18.0	Yes	0.03%
Switzerland (2014)	85 206	34.2	Yes	5.7%
UK: England	636 230	27.0	Partial^g^	(≈85%)^d^
UK: Northern Ireland	24 544	29.9	Yes	1.6%
UK: Scotland	54 513	32.5	Partial^g^	(≈22%)^d^
UK: Wales	32 338	26.1	Partial^g^	(≈50%)^d^

^a^ Rates reported in European Perinatal Health Report (EPHR): number of caesarean sections per 100 live births and stillbirths, births with missing information on mode of delivery are excluded.

^b^ Data missing on previous caesarean section.

^c^ Data will be available starting in 2017.

^d^ National data not available in Greece, estimate from WHO expert group.^20^

^e^ Data will be available starting in 2017.

^f^ Caesarean section rate updated from EPHR where 2014 data were used.

^g^ Items available but high missing data, due to the variable on ‘presentation’ in Scotland for example.

**Figure 1:**
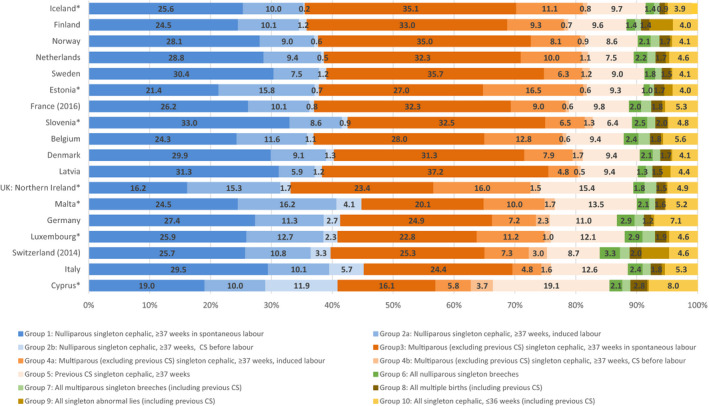
Distribution of women having live births or stillbirths by Ten‐Group Classification System group by country, ranked by overall caesarean section (CS) birth rate in 2015. Note: *at least one group has a denominator <100 women (see Tables S1‐S18).

Figure 1 compares the distribution of women having live births and stillbirths using the 12 categories in each country, ranked by their overall caesarean rate. Detailed information on the categories for each country is presented in the Supplementary material (Tables S1–S18). This distribution revealed differences between childbearing populations in their distributions by group, even when overall caesarean rates were similar. Some of the variation reflected demographic differences in the percentage of nulliparous and multiparous women: nulliparous women with term, cephalic deliveries, groups 1 and 2, ranged from about 44% in Italy, Malta and Slovenia to 36% or less in Northern Ireland, Iceland and Finland. On average, group 2 was larger in countries with higher caesarean rates (ρ = 0.62). Overall the size of group 4 was not correlated with higher caesarean rates (ρ = −0.11), but there was a strong correlation for groups 2b (ρ = 0.89) and 4b (ρ = 0.62).

The proportion of childbearing women with a previous caesarean section varied from a low of 6.4% (Slovenia) to a high of 19.1% (Cyprus). In general, the percentage of women with a previous caesarean section was higher when overall rates were higher (ρ = 0.55). Higher overall caesarean rates were also correlated with a higher proportion of births in groups 6 and 7 (breech combined) (ρ = 0.53) and 10 (singleton, cephalic preterm) (ρ = 0.69); we explored the hypothesis that a higher percentage of preterm births was related with groups 6 and 7 combined (all breech) as preterm births are more often breech; this was confirmed (ρ = 0.63). Finally, abnormal lies constituted a small proportion of births (<0.5%), except in Finland, Latvia, Estonia and Switzerland. In the first three countries this was accompanied by relatively low proportion of breeches (<2.5%), whereas in Switzerland both proportion were high.

**Figure 2:**
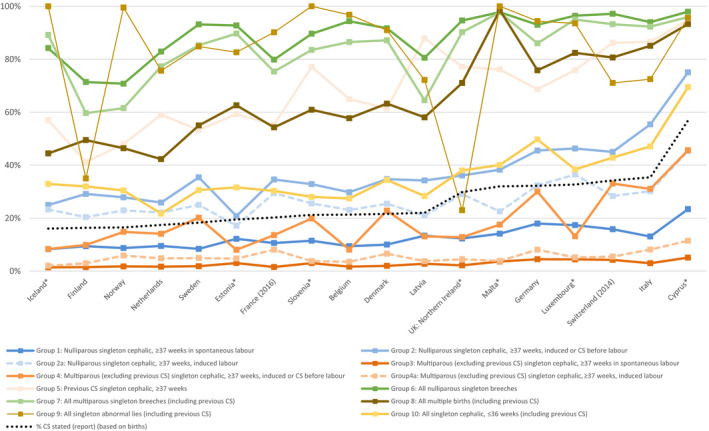
Caesarean section (CS) rate in the Ten‐Group Classification System (TGCS) groups among women delivering live or stillborn infants by country, ordered by their overall caesarean rate, in 2015. Caesarean rates in each TGCS group are presented using a different coloured marker and connected across countries by a similarly coloured line. This makes it possible to compare slopes of the line for each group with the overall caesarean rate, represented in the figure as a dotted black line, to assess concordance in country rankings. Note: *at least one group has a denominator <100 women (see Tables S1‐S18).

Figure 2 displays caesarean rates in each group by country. Countries are ordered by their overall caesarean rate and caesarean rates in each group are represented in a different colour and connected across countries by a similarly coloured line. This makes it possible to compare slopes of the line for each group with the overall caesarean rate, represented as a dotted black line. The generally increasing slopes illustrate that caesarean rates for each group, with the exception of abnormal lies, relate to the overall caesarean birth rate: range of correlations from: 0.53 for group 4a to 0.92 for group 8 (multiples). Practices were more heterogeneous for several other groups, however. For breech deliveries, for instance, caesarean rates were high in Iceland and Sweden, despite their low caesarean rates in other groups. This heterogeneity was also observed for caesarean birth after previous caesarean section; Slovenia and Denmark had different practices for this group despite having similar overall caesarean rates, whereas Germany and Belgium had similar caesarean rates after previous caesarean section, but differed in their overall caesarean rates. Some countries' practices in use of caesarean sections for singleton preterm cephalic deliveries differed from the general picture, with Germany having higher rates than countries with similar overall caesarean rates and the Netherlands having lower rates. Finally, the countries reporting a higher proportion of caesarean sections for abnormal lies had lower overall caesarean rates.

**Figure 3:**
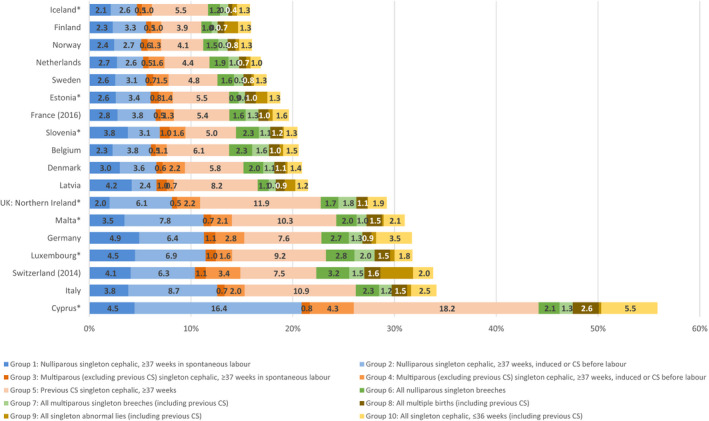
Contribution of the ten groups to the overall caesarean section (CS) rate by country ranked by overall caesarean section rate in 2015. Note: *at least one group has a denominator <100 women (see Tables S1‐S18).

In all countries, caesarean sections for women with previous caesarean sections contributed more than all other groups to the overall rate. In general, the contribution of all groups was greater in countries with higher caesarean rates, confirming the patterns observed in group‐specific caesarean rates (Figure 3). However, this picture was particularly marked for groups 2, 4 and 5. In some countries, caesarean rates in groups 1 and 2 were substantially higher than in group 5, which suggests rising caesarean rates. This figure illustrates the high contribution of breech caesarean sections in some countries, such as Belgium, Slovenia and Northern Ireland, contributing over 3 percentage points to the overall caesarean birth rate. In Germany and Cyprus, group 10, singleton preterm cephalic births, made up a higher percentage of the overall caesarean rate.

## Discussion

### Main findings

More than half of the European countries participating in the Euro‐Peristat project could classify caesarean births into the TGCS, illustrating the feasibility of using it to carry out routine monitoring of maternal and perinatal health at a national level. In general, countries with higher overall caesarean rates had higher rates in all TGCS groups. Substantial heterogeneity was also observed among countries with similar caesarean rates, however, especially for groups 5 (previous caesarean section), 6, 7 (nulliparous and multiparous breech) and 10 (singleton, preterm, cephalic). On average, countries that could not provide data on the TGCS groups had higher caesarean rates, suggesting that having more comprehensive caesarean data in routine systems might contribute to implementing evidence‐based practice.

### Strengths and limitations

The strengths of this study are the inclusion of a large number of countries, use of a common data collection protocol, crosschecks with other Euro‐Peristat indicators and the engagement of our network of experts and data providers in checking and interpreting the data. We also collected information on births with missing data, as recommended by previous reviews.^22^

Limitations primarily relate to misclassification due to unstandardised definitions and underreporting in routine data sources. These are most problematic for mode of onset of delivery, fetal presentation and previous caesarean section. For delivery onset, some countries classify caesarean sections as elective or emergency, which imperfectly maps onto whether labour did or did not begin before the caesarean section (elective caesarean sections can be carried out after labour begins and emergency caesarean sections can occur without labour). Similarly, for induction, there are inconsistencies linked to oxytocin use for induction or augmentation of labour and inclusions of amniotomy.^22^ For fetal presentation, transverse and oblique lies may not be well identified in routine statistics. In Finland, for instance, abnormal lies are defined based on a check box for both breech and other malpresentation, which is further analysed using the International Classification of Diseases tenth revision code O64, but lacks precision for identifying transverse and oblique. In other countries, transverse or oblique lies may be included in an ‘other’ category. These births should be uncommon (<0.5%) and their caesarean rates should be close to 100%, which was not the case in several countries, as observed elsewhere.^10^, ^23^ Underreporting can also occur for non‐cephalic fetal presentations and previous caesarean sections, especially if these are based on a check box or the addition of a specific code. Misclassification of mode of onset affects the ability to distinguish between groups 1 and 2(a/b) and 3 and 4(a/b), whereas underreporting of previous caesarean section and breech presentation is likely to inflate groups 4b (for both variables) and 2b (breech only). These data‐quality issues have been raised in many previous studies.^12^, ^22^ Fully documenting these differences in definitions in routine systems and investigating how they affect TGCS classification is an important area for future work.

### Interpretation

Countries with higher caesarean rates overall tended to have higher caesarean rates in all TGCS groups and a higher absolute contribution from all groups. For instance, caesarean section for multiple births ranged from under 45% in countries with low overall rates to over 95% in countries with the highest overall rates. Caesarean rates for women with a previous caesarean section were also lower (under 60%) in countries with lower caesarean rates.^8^ As others have found, differences tended to be very marked for prelabour caesarean sections, groups 2b and 4b, which are much more common in countries with high caesarean rates than in those with low rates.^8^, ^10^ However, heterogeneity was observed in specific groups even when overall caesarean rates were similar, as found in previous cross‐country comparisons.^11^ For instance, Slovenia had a higher caesarean rate in group 5 (previous caesarean section) than other countries with similar overall caesarean rates. The proportion of nulliparous births with induced labour also varied. For instance, it was lower in Sweden, perhaps reflecting greater tolerance for post‐term births.^24^ Increases in groups of inductions have been noted over time in many studies, but the impact on the overall rates of caesarean birth is not clear.^11^ As our study includes only one time‐point, we are unable to analyse trends over time unlike previous national‐level studies.^8^, ^10^, ^11^, ^25^

Another heterogeneous group was breech deliveries. Most countries with high caesarean rates had breech delivery caesarean rates of 90% and over, as also reported in the USA,^10^ but practices were more heterogeneous in countries with low caesarean rates, being low in France and higher in Sweden, for example. In France, vaginal delivery for breech presentation continues to be considered an option after a large population‐based study found good outcomes when strict protocols were observed.^26^ Finally, although the caesarean rates for group 10 (singleton preterm cephalic births) increased in tandem with the overall caesarean rate, some countries stood out, such as Germany with high rates, leading to a contribution of 3.5% in absolute terms. This is consistent with other research showing high caesarean rates for preterm birth in Germany.^27^, ^28^

### Policy and practice

While the TGCS was primarily conceived as a tool to assess clinical practice at the facility level, it is useful for national and international reporting because it facilitates comparisons between homogeneous clinical groups and accentuates how caesarean sections in first pregnancies contribute to increasing caesarean rates. However, although these analyses constitute a first step towards investigating differences in caesarean rates,^6^ they do not elucidate the underlying reasons for these differences. Some studies have adjusted for risk factors, such as maternal age, body mass index or comorbidities in order to separate changes in population case‐mix from change in practice.^25^, ^29^ This would be useful in future European analyses as there are wide differences in parity and maternal age distributions. For instance, the percentage of women aged 35 years and over in Euro‐Peristat countries ranges from 13.6 to 37.3%.^1^

How care is organised, including the financial and time management incentives for obstetricians to perform caesarean sections as well as the availability of midwives and the nature of their roles within maternity services, is likely to be a central reason for many of these differences.^2^, ^3^, ^4^ Information on the indication for the caesarean section could also provide more insight into practice differences.^30^ Classifying indications within TCGS groups could improve the usefulness of indication data, which are difficult to compare because of high variability in definitions and classifications.^31^ Finally, assessing the impact of cross‐country differences requires analysis of maternal and neonatal outcomes alongside the caesarean rate in each group.

Unfortunately, we could not include many of the countries in Europe with the highest caesarean rates in this study because they were unable to construct the TGCS. In the 2015 Euro‐Peristat report, these countries accounted for most of those with a caesarean birth rate of over 30% and had increases over 10% between 2010 and 2015.^15^ There is a need to increase the range, completeness and quality of the data collected and to develop national data collection systems so that the best use can be made of the data they contain.

## Conclusion

We were able to construct the TGCS for over half of European countries. Future work should continue to harmonise data definitions, and this classification constitutes an important comparator for individual countries and hospitals within these countries, baseline data for future surveillance and an impetus for countries to improve the quality and scope of their data systems.

### Disclosure of interests

The authors have no interests to disclose. Completed disclosure of interests forms are available to view online as supporting information.

### Contribution to authorship

JZ, MD, AM, SA, GH, ML, JN, HSO, EM, MG and BB conceived the study. JZ, MD and BB drafted the first draft and were responsible for the analysis. AM, GH, ML, JN, HSO, EM and MG contributed to analysis and interpretation of data. JZ, MD, AM, SA, GH, ML, JN, HSO, EM, MG, BB and the Euro‐Peristat Network critically reviewed and revised the manuscript. JZ, MD, AM, SA, GH, ML, JN, HSO, EM, MG, BB and the Euro‐Peristat Network provided data for the study and participated in interpretation. JZ, MD, AM, SA, GH, ML, JN, HSO, EM, MG, BB and the Euro‐Peristat Network approved the final manuscript.

### Details of ethics approval

Euro‐Peristat received authorisations for its 2015 core database from the French Advisory Committee on Use of Health Data in Medical Research (N°17‐048, 30 March 2017) and the French National Commission for Data Protection and Liberties (DR‐2019‐089, 26 March 2019).

### Funding

InfAct Joint Action (Grant no. 801553) and Bridge Health Project (Grant no. 665691), Public Health Programme, Consumers, Health, Agriculture and Food Executive Agency (CHAFEA).

## Supporting information

**Table S1.** Robson's Ten‐Group Classification System, including sub‐groups, in Belgium 2015.
**Table S2.** Robson's Ten‐Group Classification System, including sub‐groups, in Cyprus 2015.
**Table S3.** Robson's Ten‐Group Classification System, including sub‐groups, in Denmark 2015.
**Table S4.** Robson's Ten‐Group Classification System, including sub‐groups, in Estonia 2015.
**Table S5.** Robson's Ten‐Group Classification System, including sub‐groups, in Finland 2015.
**Table S6.** Robson's Ten‐Group Classification System, including sub‐groups, in France 2016.
**Table S7.** Robson's Ten‐Group Classification System, including sub‐groups, in Germany 2015.
**Table S8.** Robson's Ten‐Group Classification System, including sub‐groups, in Iceland 2015.
**Table S9.** Robson's Ten‐Group Classification System, including sub‐groups, in Italy 2015.
**Table S10.** Robson's Ten‐Group Classification System, including sub‐groups, in Latvia 2015.
**Table S11.** Robson's Ten‐Group Classification System, including sub‐groups, in Luxembourg 2015.
**Table S12.** Robson's Ten‐Group Classification System, including sub‐groups, in Malta 2015.
**Table S13.** Robson's Ten‐Group Classification System, including sub‐groups, in the Netherlands 2015.
**Table S14.** Robson's Ten‐Group Classification System, including sub‐groups, in Northern Ireland 2015.
**Table S15.** Robson's Ten‐Group Classification System, including sub‐groups, in Norway 2015.
**Table S16.** Robson's Ten‐Group Classification System, including sub‐groups, in Slovenia 2015.
**Table S17.** Robson's Ten‐Group Classification System, including sub‐groups, in Sweden 2015.
**Table S18.** Robson's Ten‐Group Classification System, including sub‐groups, in Switzerland 2014.

Supplementary Material

Supplementary Material

Supplementary Material

Supplementary Material

Supplementary Material

Supplementary Material

Supplementary Material

Supplementary Material

Supplementary Material

Supplementary Material

Supplementary Material

## Data Availability

The data are provided as an online supplement to the manuscript.
