# Supplementary material for: Using Robson's Ten‐Group Classification System for comparing caesarean section rates in Europe: an analysis of routine data from the Euro‐Peristat study
Source: BJOG. 2021 Feb 1;128(9):1444–53. doi: 10.1111/1471-0528.16634 (PMC8359161; doi:10.1111/1471-0528.16634)
Supplement: Supplementary file 1 — Table S1. Robson's Ten‐Group Classification System, including sub‐groups, in Belgium 2015. Table S2. Robson's Ten‐Group Classification System, including sub‐groups, in Cyprus 2015. Table S3. Robson's Ten‐Group Classification System, including sub‐groups, in Denmark 2015. Table S4. Robson's Ten‐Group Classification System, including sub‐groups, in Estonia 2015. Table S5. Robson's Ten‐Group Classification System, including sub‐groups, in Finland 2015. Table S6. Robson's Ten‐Group Classification System, including sub‐groups, in France 2016. Table S7. Robson's Ten‐Group Classification System, including sub‐groups, in Germany 2015. Table S8. Robson's Ten‐Group Classification System, including sub‐groups, in Iceland 2015. Table S9. Robson's Ten‐Group Classification System, including sub‐groups, in Italy 2015. Table S10. Robson's Ten‐Group Classification System, including sub‐groups, in Latvia 2015. Table S11. Robson's Ten‐Group Classification System, including sub‐groups, in Luxembourg 2015. Table S12. Robson's Ten‐Group Classification System, including sub‐groups, in Malta 2015. Table S13. Robson's Ten‐Group Classification System, including sub‐groups, in the Netherlands 2015. Table S14. Robson's Ten‐Group Classification System, including sub‐groups, in Northern Ireland 2015. Table S15. Robson's Ten‐Group Classification System, including sub‐groups, in Norway 2015. Table S16. Robson's Ten‐Group Classification System, including sub‐groups, in Slovenia 2015. Table S17. Robson's Ten‐Group Classification System, including sub‐groups, in Sweden 2015. Table S18. Robson's Ten‐Group Classification System, including sub‐groups, in Switzerland 2014. [file BJO-128-1444-s004.pdf]

Table S1. Robson's Ten Group Classification System, including sub-groups, in Belgium 2015

	Number of CS/ all women in each group	Number of CS/ all women in each group imputed data	Relative size of each group (%)	CS rate in each group (%)	Contribution of each group (%)
Group 1: Nullip single ceph ≥ 37 wks spon lab	2710/28844	2760/29356	24.3	9.4	2.3
Group 2a: Nullip single ceph ≥ 37 wks induced	3165/13719	3224/13965	11.6	23.1	2.7
Group 2b: Nullip single ceph ≥ 37 wks CS before lab	1306/1306	1330/1330	1.1	100.0	1.1
Group 3: Multip (excl prev caesarean sections) single ceph ≥ 37 wks spon lab	559/33236	569/33824	28.0	1.7	0.5
Group 4a: Multip (excl prev caesarean sections) single ceph ≥ 37 wks induced	533/15234	543/15504	12.8	3.5	0.4
Group 4b: Multip (excl prev caesarean sections) single ceph ≥ 37 wks CS before lab	761/761	775/775	0.6	100.0	0.6
Group 5: Previous caesarean section single ceph ≥ 37 wks	7255/11177	7389/11380	9.4	64.9	6.1
Group 6: All nulliparous, single breeches	2695/2856	2745/2909	2.4	94.4	2.3
Group 7: All multiparous, single breeches (incl previous caesarean sections)	1939/2242	1975/2283	1.9	86.5	1.6
Group 8: All multiple births (incl previous caesarean sections)	1235/2140	1258/2179	1.8	57.7	1.0
Group 9: All single abnormal lies (incl previous caesarean sections)	412/426	420/434	0.4	96.8	0.3
Group 10: All single ceph ≤ 36 wks (incl previous caesarean sections)	1820/6641	1854/6760	5.6	27.4	1.5
Not stated	217/2116		-	-	-
**Total**			**100.0**		**20.6**

Note: missing data are imputed using the observed distributions for caesarean births, then for non-caesarean births. Because of the low proportion of missing data for caesareans, we first imputed the missing data into caesarean/non-caesarean using the overall caesarean rate.

Table S2. Robson's Ten Group Classification System, including sub-groups, in Cyprus 2015

	Number of CS/ all women in each group	Number of CS/ all women in each group imputed data	Relative size of each group (%)	CS rate in each group (%)	Contribution of each group (%)
Group 1: Nullip single ceph ≥ 37 wks spon lab	403/1719	409/1748	19.0	23.4	4.5
Group 2a: Nullip single ceph ≥ 37 wks induced	409/901	415/916	10.0	45.3	4.5
Group 2b: Nullip single ceph ≥ 37 wks CS before lab	1076/1076	1091/1091	11.9	100.0	11.9
Group 3: Multip (excl prev caesarean sections) single ceph ≥ 37 wks spon lab	74/1449	75/1474	16.1	5.1	0.8
Group 4a: Multip (excl prev caesarean sections) single ceph ≥ 37 wks induced	60/524	61/533	5.8	11.4	0.7
Group 4b: Multip (excl prev caesarean sections) single ceph ≥ 37 wks CS before lab	330/330	335/335	3.7	100.0	3.7
Group 5: Previous caesarean section single ceph ≥ 37 wks	1643/1731	1666/1756	19.1	94.9	18.2
Group 6: All nulliparous, single breeches	187/191	190/194	2.1	97.9	2.1
Group 7: All multiparous, single breeches (incl previous caesarean sections)	116/121	118/123	1.3	95.9	1.3
Group 8: All multiple births (incl previous caesarean sections)	233/250	236/253	2.8	93.3	2.6
Group 9: All single abnormal lies (incl previous caesarean sections)	22/23	22/23	0.3	95.7	0.2
Group 10: All single ceph ≤ 36 wks (incl previous caesarean sections)	500/719	507/730	8.0	69.5	5.5
Not stated	72/141		-	-	-
**Total**			**100.0**		**55.9**

Note: missing data are imputed using the observed distributions for caesarean births, then for non-caesarean births.

Table S3. Robson's Ten Group Classification System, including sub-groups, in Denmark 2015

	Number of CS/ all women in each group	Number of CS/ all women in each group imputed data	Relative size of each group (%)	CS rate in each group (%)	Contribution of each group (%)
Group 1: Nullip single ceph ≥ 37 wks spon lab	1699/16951	1712/17101	29.9	10.0	3.0
Group 2a: Nullip single ceph ≥ 37 wks induced	1307/5134	1317/5178	9.1	25.4	2.3
Group 2b: Nullip single ceph ≥ 37 wks CS before lab	730/730	736/736	1.3	100.0	1.3
Group 3: Multip (excl prev caesarean sections) single ceph ≥ 37 wks spon lab	348/17703	351/17862	31.3	2.0	0.6
Group 4a: Multip (excl prev caesarean sections) single ceph ≥ 37 wks induced	296/4495	298/4535	7.9	6.6	0.5
Group 4b: Multip (excl prev caesarean sections) single ceph ≥ 37 wks CS before lab	948/948	956/956	1.7	100.0	1.7
Group 5: Previous caesarean section single ceph ≥ 37 wks	3259/5303	3285/5347	9.4	61.4	5.8
Group 6: All nulliparous, single breeches	1107/1207	1116/1217	2.1	91.7	2.0
Group 7: All multiparous, single breeches (incl previous caesarean sections)	612/702	617/708	1.2	87.1	1.1
Group 8: All multiple births (incl previous caesarean sections)	616/974	621/982	1.7	63.2	1.1
Group 9: All single abnormal lies (incl previous caesarean sections)	120/132	121/133	0.2	91.0	0.2
Group 10: All single ceph ≤ 36 wks (incl previous caesarean sections)	803/2331	809/2351	4.1	34.4	1.4
Not stated	94/497		-	-	-
**Total**			**100.0**		**20.9**

Note: missing data are imputed using the observed distributions for caesarean births, then for non-caesarean births.

Table S4. Robson's Ten Group Classification System, including sub-groups, in Estonia 2015

	Number of CS/ all women in each group	Number of CS/ all women in each group imputed data	Relative size of each group (%)	CS rate in each group (%)	Contribution of each group (%)
Group 1: Nullip single ceph ≥ 37 wks spon lab	357/2932	357/2932	21.4	12.2	2.6
Group 2a: Nullip single ceph ≥ 37 wks induced	371/2167	371/2167	15.8	17.1	2.7
Group 2b: Nullip single ceph ≥ 37 wks CS before lab	100/100	100/100	0.7	100.0	0.7
Group 3: Multip (excl prev caesarean sections) single ceph ≥ 37 wks spon lab	112/3701	112/3701	27.0	3.0	0.8
Group 4a: Multip (excl prev caesarean sections) single ceph ≥ 37 wks induced	107/2260	107/2260	16.5	4.7	0.8
Group 4b: Multip (excl prev caesarean sections) single ceph ≥ 37 wks CS before lab	81/81	81/81	0.6	100.0	0.6
Group 5: Previous caesarean section single ceph ≥ 37 wks	759/1281	759/1281	9.3	59.3	5.5
Group 6: All nulliparous, single breeches	128/138	128/138	1.0	92.8	0.9
Group 7: All multiparous, single breeches (incl previous caesarean sections)	87/97	87/97	0.7	89.7	0.6
Group 8: All multiple births (incl previous caesarean sections)	144/230	144/230	1.7	62.6	1.0
Group 9: All single abnormal lies (incl previous caesarean sections)	157/190	157/190	1.4	82.6	1.1
Group 10: All single ceph ≤ 36 wks (incl previous caesarean sections)	175/554	175/554	4.0	31.6	1.3
Not stated	0/1	-	-	-	-
**Total**			**100.0**		**18.8**

Note: missing data are imputed using the observed distributions for caesarean births, then for non-caesarean births.

Table S5. Robson's Ten Group Classification System, including sub-groups, in Finland 2015

	Number of CS/ all women in each group	Number of CS/ all women in each group imputed data	Relative size of each group (%)	CS rate in each group (%)	Contribution of each group (%)
Group 1: Nullip single ceph ≥ 37 wks spon lab	1267/13484	1268/13501	24.5	9.4	2.3
Group 2a: Nullip single ceph ≥ 37 wks induced	1127/5525	1128/5532	10.1	20.4	2.1
Group 2b: Nullip single ceph ≥ 37 wks CS before lab	681/681	682/682	1.2	100.0	1.2
Group 3: Multip (excl prev caesarean sections) single ceph ≥ 37 wks spon lab	269/18104	269/18127	33.0	1.5	0.5
Group 4a: Multip (excl prev caesarean sections) single ceph ≥ 37 wks induced	151/5125	151/5131	9.3	2.9	0.3
Group 4b: Multip (excl prev caesarean sections) single ceph ≥ 37 wks CS before lab	394/394	394/394	0.7	100.0	0.7
Group 5: Previous caesarean section single ceph ≥ 37 wks	2161/5250	2163/5256	9.6	41.2	3.9
Group 6: All nulliparous, single breeches	561/786	562/787	1.4	71.4	1.0
Group 7: All multiparous, single breeches (incl previous caesarean sections)	318/533	318/533	1.0	59.7	0.6
Group 8: All multiple births (incl previous caesarean sections)	370/748	370/748	1.4	49.5	0.7
Group 9: All single abnormal lies (incl previous caesarean sections)	743/2124	744/2127	3.9	35.0	1.4
Group 10: All single ceph ≤ 36 wks (incl previous caesarean sections)	700/2190	701/2193	4.0	32.0	1.3
Not stated	8/67	-	-	-	-
**Total**			**100.0**		**15.9**

Note: missing data are imputed using the observed distributions for caesarean births, then for non-caesarean births.

Table S6. Robson's Ten Group Classification System, including sub-groups, in France 2016

	Number of CS/ all women in each group	Number of CS/ all women in each group imputed data	Relative size of each group (%)	CS rate in each group (%)	Contribution of each group (%)
Group 1: Nullip single ceph ≥ 37 wks spon lab	359/3412	363/3427	26.2	10.6	2.8
Group 2a: Nullip single ceph ≥ 37 wks induced	383/1309	388/1317	10.1	29.5	3.0
Group 2b: Nullip single ceph ≥ 37 wks CS before lab	102/102	103/103	0.8	100.0	0.8
Group 3: Multip (excl prev caesarean sections) single ceph ≥ 37 wks spon lab	64/4210	65/4226	32.3	1.5	0.5
Group 4a: Multip (excl prev caesarean sections) single ceph ≥ 37 wks induced	94/1172	95/1177	9.0	8.1	0.7
Group 4b: Multip (excl prev caesarean sections) single ceph ≥ 37 wks CS before lab	74/74	75/75	0.6	100.0	0.6
Group 5: Previous caesarean section single ceph ≥ 37 wks	703/1276	711/1286	9.8	55.3	5.4
Group 6: All nulliparous, single breeches	208/261	210/263	2.0	79.8	1.6
Group 7: All multiparous, single breeches (incl previous caesarean sections)	163/217	165/219	1.7	75.3	1.3
Group 8: All multiple births (incl previous caesarean sections)	126/233	127/234	1.8	54.3	1.0
Group 9: All single abnormal lies (incl previous caesarean sections)	54/60	55/61	0.5	90.2	0.4
Group 10: All single ceph ≤ 36 wks (incl previous caesarean sections)	207/686	209/690	5.3	30.3	1.6
Not stated	30/67	-	-	-	-
**Total**			**100.0**		**19.6**

Note: missing data are imputed using the observed distributions for caesarean births, then for non-caesarean births.

Table S7. Robson's Ten Group Classification System, including sub-groups, in Germany 2015

	Number of CS/ all women in each group	Number of CS/ all women in each group imputed data	Relative size of each group (%)	CS rate in each group (%)	Contribution of each group (%)
Group 1: Nullip single ceph ≥ 37 wks spon lab	35357/196968	35359/197013	27.4	17.9	4.9
Group 2a: Nullip single ceph ≥ 37 wks induced	26274/81111	26275/81127	11.3	32.4	3.6
Group 2b: Nullip single ceph ≥ 37 wks CS before lab	19545/19545	19546/19546	2.7	100.0	2.7
Group 3: Multip (excl prev caesarean sections) single ceph ≥ 37 wks spon lab	8038/179511	8038/179557	24.9	4.5	1.1
Group 4a: Multip (excl prev caesarean sections) single ceph ≥ 37 wks induced	4205/51987	4205/52000	7.2	8.1	0.6
Group 4b: Multip (excl prev caesarean sections) single ceph ≥ 37 wks CS before lab	16285/16285	16286/16286	2.3	100.0	2.3
Group 5: Previous caesarean section single ceph ≥ 37 wks	54548/79447	54551/79457	11.0	68.7	7.6
Group 6: All nulliparous, single breeches	19417/20875	19418/20876	2.9	93.0	2.7
Group 7: All multiparous, single breeches (incl previous caesarean sections)	9557/11111	9557/11111	1.5	86.0	1.3
Group 8: All multiple births (incl previous caesarean sections)	6479/8544	6479/8545	1.2	75.8	0.9
Group 9: All single abnormal lies (incl previous caesarean sections)	3260/3454	3260/3454	0.5	94.4	0.5
Group 10: All single ceph ≤ 36 wks (incl previous caesarean sections)	25350/50973	25351/50981	7.1	49.7	3.5
Not stated	11/142	-	-	-	-
**Total**			**100.0**		**31.7**

Note: missing data are imputed using the observed distributions for caesarean births, then for non-caesarean births.

Table S8. Robson’s Ten Group Classification System, including sub-groups, in Iceland 2015

	Number of CS/ all women in each group	Number of CS/ all women in each group imputed data	Relative size of each group (%)	CS rate in each group (%)	Contribution of each group (%)
Group 1: Nullip single ceph ≥ 37 wks spon lab	86/1021	86/1041	25.6	8.3	2.1
Group 2a: Nullip single ceph ≥ 37 wks induced	95/401	95/408	10.0	23.3	2.3
Group 2b: Nullip single ceph ≥ 37 wks CS before lab	9/9	9/9	0.2	100.0	0.2
Group 3: Multip (excl prev caesarean sections) single ceph ≥ 37 wks spon lab	20/1395	20/1425	35.1	1.4	0.5
Group 4a: Multip (excl prev caesarean sections) single ceph ≥ 37 wks induced	9/440	9/449	11.1	2.0	0.2
Group 4b: Multip (excl prev caesarean sections) single ceph ≥ 37 wks CS before lab	31/31	31/31	0.8	100.0	0.8
Group 5: Previous caesarean section single ceph ≥ 37 wks	224/389	224/393	9.7	57.0	5.5
Group 6: All nulliparous, single breeches	48/57	48/57	1.4	84.2	1.2
Group 7: All multiparous, single breeches (incl previous caesarean sections)	33/37	33/37	0.9	89.2	0.8
Group 8: All multiple births (incl previous caesarean sections)	16/36	16/36	0.9	44.4	0.4
Group 9: All single abnormal lies (incl previous caesarean sections)	19/19	19/19	0.5	100.0	0.5
Group 10: All single ceph ≤ 36 wks (incl previous caesarean sections)	52/156	52/158	3.9	32.9	1.3
Not stated	0/72	-	-	-	-
**Total**			**100.0**		**15.8**

Note: missing data are imputed using the observed distributions for caesarean births, then for non-caesarean births.

Table S9. Robson's Ten Group Classification System, including sub-groups, in Italy 2015

	**Number of CS/ all women in each group**	**Number of CS/ all women in each group imputed data**	**Relative size of each group (%)**	**CS rate in each group (%)**	**Contribution of each group (%)**
Group 1: Nullip single ceph ≥ 37 wks spon lab	17321/137708	18409/140974	29.5	13.1	3.8
Group 2a: Nullip single ceph ≥ 37 wks induced	13675/46694	14534/48150	10.1	30.2	3.0
Group 2b: Nullip single ceph ≥ 37 wks CS before lab	25626/25626	27236/27236	5.7	100.0	5.7
Group 3: Multip (excl prev caesarean sections) single ceph ≥ 37 wks spon lab	3274/114610	3480/116830	24.4	3.0	0.7
Group 4a: Multip (excl prev caesarean sections) single ceph ≥ 37 wks induced	1742/22248	1851/22728	4.8	8.1	0.4
Group 4b: Multip (excl prev caesarean sections) single ceph ≥ 37 wks CS before lab	7098/7098	7544/7544	1.6	100.0	1.6
Group 5: Previous caesarean section single ceph ≥ 37 wks	49193/57165	52283/60399	12.6	86.6	10.9
Group 6: All nulliparous, single breeches	10287/10974	10933/11632	2.4	94.0	2.3
Group 7: All multiparous, single breeches (incl previous caesarean sections)	5547/6029	5895/6386	1.3	92.3	1.2
Group 8: All multiple births (incl previous caesarean sections)	6899/8163	7332/8619	1.8	85.1	1.5
Group 9: All single abnormal lies (incl previous caesarean sections)	1718/2400	1826/2520	0.5	72.5	0.4
Group 10: All single ceph ≤ 36 wks (incl previous caesarean sections)	11243/24430	11949/25375	5.3	47.1	2.5
Not stated	9651/15250		-	-	-
**Total**			**100.0**		**34.1**

Note: missing data are imputed using the observed distributions for caesarean births, then for non-caesarean births.

Table S10. Robson's Ten Group Classification System, including sub-groups, in Latvia 2015

	Number of CS/ all women in each group	Number of CS/ all women in each group imputed data	Relative size of each group (%)	CS rate in each group (%)	Contribution of each group (%)
Group 1: Nullip single ceph ≥ 37 wks spon lab	898/6721	898/6721	31.3	13.4	4.2
Group 2a: Nullip single ceph ≥ 37 wks induced	267/1270	267/1270	5.9	21.0	1.2
Group 2b: Nullip single ceph ≥ 37 wks CS before lab	255/255	255/255	1.2	100.0	1.2
Group 3: Multip (excl prev caesarean sections) single ceph ≥ 37 wks spon lab	220/7994	220/7994	37.2	2.8	1.0
Group 4a: Multip (excl prev caesarean sections) single ceph ≥ 37 wks induced	39/1036	39/1036	4.8	3.8	0.2
Group 4b: Multip (excl prev caesarean sections) single ceph ≥ 37 wks CS before lab	111/111	111/111	0.5	100.0	0.5
Group 5: Previous caesarean section single ceph ≥ 37 wks	1769/2012	1769/2012	9.4	87.9	8.2
Group 6: All nulliparous, single breeches	232/288	232/288	1.3	80.6	1.1
Group 7: All multiparous, single breeches (incl previous caesarean sections)	149/231	149/231	1.1	64.5	0.7
Group 8: All multiple births (incl previous caesarean sections)	191/329	191/329	1.5	58.1	0.9
Group 9: All single abnormal lies (incl previous caesarean sections)	223/309	223/309	1.4	72.2	1.0
Group 10: All single ceph ≤ 36 wks (incl previous caesarean sections)	267/942	267/942	4.4	28.3	1.2
Not stated	0/0		-	-	-
**Total**			**100.0**		**21.5**

Note: missing data are imputed using the observed distributions for caesarean births, then for non-caesarean births.

Table S11. Robson's Ten Group Classification System, including sub-groups, in Luxembourg 2015

	**Number of CS/ all women in each group**	**Number of CS/ all women in each group imputed data**	**Relative size of each group (%)**	**CS rate in each group (%)**	**Contribution of each group (%)**
Group 1: Nullip single ceph ≥ 37 wks spon lab	303/1747	303/1747	25.9	17.3	4.5
Group 2a: Nullip single ceph ≥ 37 wks induced	311/853	311/853	12.7	36.5	4.6
Group 2b: Nullip single ceph ≥ 37 wks CS before lab	156/156	156/156	2.3	100.0	2.3
Group 3: Multip (excl prev caesarean sections) single ceph ≥ 37 wks spon lab	68/1539	68/1539	22.8	4.4	1.0
Group 4a: Multip (excl prev caesarean sections) single ceph ≥ 37 wks induced	39/752	39/752	11.2	5.2	0.6
Group 4b: Multip (excl prev caesarean sections) single ceph ≥ 37 wks CS before lab	69/69	69/69	1.0	100.0	1.0
Group 5: Previous caesarean section single ceph ≥ 37 wks	620/817	621/818	12.1	75.9	9.2
Group 6: All nulliparous, single breeches	189/196	189/196	2.9	96.4	2.8
Group 7: All multiparous, single breeches (incl previous caesarean sections)	133/140	133/140	2.1	95.0	2.0
Group 8: All multiple births (incl previous caesarean sections)	103/125	103/125	1.9	82.4	1.5
Group 9: All single abnormal lies (incl previous caesarean sections)	29/31	29/31	0.5	93.5	0.4
Group 10: All single ceph ≤ 36 wks (incl previous caesarean sections)	119/310	119/310	4.6	38.4	1.8
Not stated	2/3	-	-	-	-
**Total**			**100.0**		**31.8**

Note: missing data are imputed using the observed distributions for caesarean births, then for non-caesarean births.

Table S12. Robson's Ten Group Classification System, including sub-groups, in Malta 2015

	Number of CS/ all women in each group	Number of CS/ all women in each group imputed data	Relative size of each group (%)	CS rate in each group (%)	Contribution of each group (%)
Group 1: Nullip single ceph ≥ 37 wks spon lab	152/1076	152/1076	24.5	14.1	3.5
Group 2a: Nullip single ceph ≥ 37 wks induced	160/710	160/710	16.2	22.5	3.6
Group 2b: Nullip single ceph ≥ 37 wks CS before lab	181/181	181/181	4.1	100.0	4.1
Group 3: Multip (excl prev caesarean sections) single ceph ≥ 37 wks spon lab	32/880	32/880	20.1	3.6	0.7
Group 4a: Multip (excl prev caesarean sections) single ceph ≥ 37 wks induced	17/440	17/440	10.0	3.9	0.4
Group 4b: Multip (excl prev caesarean sections) single ceph ≥ 37 wks CS before lab	73/73	73/73	1.7	100.0	1.7
Group 5: Previous caesarean section single ceph ≥ 37 wks	450/591	450/591	13.5	76.1	10.3
Group 6: All nulliparous, single breeches	88/90	88/90	2.1	97.8	2.0
Group 7: All multiparous, single breeches (incl previous caesarean sections)	42/43	42/43	1.0	97.7	1.0
Group 8: All multiple births (incl previous caesarean sections)	67/68	67/68	1.6	98.5	1.5
Group 9: All single abnormal lies (incl previous caesarean sections)	7/7	7/7	0.2	100.0	0.2
Group 10: All single ceph ≤ 36 wks (incl previous caesarean sections)	91/227	91/227	5.2	40.1	2.1
Not stated	0/0	-	-	-	-
**Total**			**100.0**		**31.0**

Note: missing data are imputed using the observed distributions for caesarean births, then for non-caesarean births.

Table S13. Robson's Ten Group Classification System, including sub-groups, in Netherlands 2015

	Number of CS/ all women in each group	Number of CS/ all women in each group imputed data	Relative size of each group (%)	CS rate in each group (%)	Contribution of each group (%)
Group 1: Nullip single ceph ≥ 37 wks spon lab	4401/46481	4540/47891	28.8	9.5	2.7
Group 2a: Nullip single ceph ≥ 37 wks induced	3371/15259	3477/15724	9.4	22.1	2.1
Group 2b: Nullip single ceph ≥ 37 wks CS before lab	773/773	797/797	0.5	100.0	0.5
Group 3: Multip (excl prev caesarean sections) single ceph ≥ 37 wks spon lab	878/52277	906/53858	32.3	1.7	0.5
Group 4a: Multip (excl prev caesarean sections) single ceph ≥ 37 wks induced	777/16155	802/16645	10.0	4.8	0.5
Group 4b: Multip (excl prev caesarean sections) single ceph ≥ 37 wks CS before lab	1744/1744	1799/1799	1.1	100.0	1.1
Group 5: Previous caesarean section single ceph ≥ 37 wks	7134/12094	7359/12469	7.5	59.0	4.4
Group 6: All nulliparous, single breeches	3006/3628	3101/3742	2.2	82.9	1.9
Group 7: All multiparous, single breeches (incl previous caesarean sections)	1691/2188	1744/2256	1.4	77.3	1.0
Group 8: All multiple births (incl previous caesarean sections)	1134/2685	1170/2768	1.7	42.3	0.7
Group 9: All single abnormal lies (incl previous caesarean sections)	679/897	700/925	0.6	75.7	0.4
Group 10: All single ceph ≤ 36 wks (incl previous caesarean sections)	1614/7448	1665/7675	4.6	21.7	1.0
Not stated	497/4920	-	-	-	-
**Total**			**100.0**		**16.8**

Note: missing data are imputed using the observed distributions for caesarean births, then for non-caesarean births. Because of the low proportion of missing data for caesareans, we first imputed the missing data into caesarean/non-caesarean using the overall caesarean rate.

Table S14. Robson's Ten Group Classification System, including sub-groups, in Northern Ireland 2015

	Number of CS/ all women in each group	Number of CS/ all women in each group imputed data	Relative size of each group (%)	CS rate in each group (%)	Contribution of each group (%)
Group 1: Nullip single ceph ≥ 37 wks spon lab	466/3879	483/3922	16.2	12.3	2.0
Group 2a: Nullip single ceph ≥ 37 wks induced	1037/3644	1075/3702	15.3	29.0	4.4
Group 2b: Nullip single ceph ≥ 37 wks CS before lab	395/395	410/410	1.7	100.0	1.7
Group 3: Multip (excl prev caesarean sections) single ceph ≥ 37 wks spon lab	119/5625	123/5670	23.4	2.2	0.5
Group 4a: Multip (excl prev caesarean sections) single ceph ≥ 37 wks induced	167/3831	173/3865	16.0	4.5	0.7
Group 4b: Multip (excl prev caesarean sections) single ceph ≥ 37 wks CS before lab	357/357	370/370	1.5	100.0	1.5
Group 5: Previous caesarean section single ceph ≥ 37 wks	2768/3610	2870/3718	15.4	77.2	11.9
Group 6: All nulliparous, single breeches	407/431	422/446	1.8	94.6	1.7
Group 7: All multiparous, single breeches (incl previous caesarean sections)	410/456	425/471	1.9	90.2	1.8
Group 8: All multiple births (incl previous caesarean sections)	251/356	260/366	1.5	71.0	1.1
Group 9: All single abnormal lies (incl previous caesarean sections)	16/73	17/74	0.3	23.0	0.1
Group 10: All single ceph ≤ 36 wks (incl previous caesarean sections)	432/1158	448/1179	4.9	38.0	1.9
Not stated	252/380	-	-	-	-
**Total**			**100.0**		**29.2**

Note: missing data are imputed using the observed distributions for caesarean births, then for non-caesarean births.

Table S15. Robson's Ten Group Classification System, including sub-groups, in Norway 2015

	Number of CS/ all women in each group	Number of CS/ all women in each group imputed data	Relative size of each group (%)	CS rate in each group (%)	Contribution of each group (%)
Group 1: Nullip single ceph ≥ 37 wks spon lab	1434/16511	1441/16572	28.1	8.7	2.4
Group 2a: Nullip single ceph ≥ 37 wks induced	1207/5263	1213/5284	9.0	23.0	2.1
Group 2b: Nullip single ceph ≥ 37 wks CS before lab	360/366	362/368	0.6	98.4	0.6
Group 3: Multip (excl prev caesarean sections) single ceph ≥ 37 wks spon lab	362/20524	364/20598	35.0	1.8	0.6
Group 4a: Multip (excl prev caesarean sections) single ceph ≥ 37 wks induced	279/4757	280/4774	8.1	5.9	0.5
Group 4b: Multip (excl prev caesarean sections) single ceph ≥ 37 wks CS before lab	499/501	502/504	0.9	99.6	0.9
Group 5: Previous caesarean section single ceph ≥ 37 wks	2426/5058	2438/5079	8.6	48.0	4.1
Group 6: All nulliparous, single breeches	872/1233	876/1238	2.1	70.8	1.5
Group 7: All multiparous, single breeches (incl previous caesarean sections)	531/864	534/868	1.5	61.5	0.9
Group 8: All multiple births (incl previous caesarean sections)	463/998	465/1002	1.7	46.4	0.8
Group 9: All single abnormal lies (incl previous caesarean sections)	204/205	205/206	0.3	99.5	0.3
Group 10: All single ceph ≤ 36 wks (incl previous caesarean sections)	738/2427	742/2437	4.1	30.4	1.3
Not stated	48/225	-	-	-	-
**Total**			**100.0**		**16.0**

Note: missing data are imputed using the observed distributions for caesarean births, then for non-caesarean births.

Table S16. Robson's Ten Group Classification System, including sub-groups, in Slovenia 2015

	Number of CS/ all women in each group	Number of CS/ all women in each group imputed data	Relative size of each group (%)	CS rate in each group (%)	Contribution of each group (%)
Group 1: Nullip single ceph ≥ 37 wks spon lab	752/6574	755/6583	33.0	11.5	3.8
Group 2a: Nullip single ceph ≥ 37 wks induced	437/1716	439/1719	8.6	25.5	2.2
Group 2b: Nullip single ceph ≥ 37 wks CS before lab	186/186	187/187	0.9	100.0	0.9
Group 3: Multip (excl prev caesarean sections) single ceph ≥ 37 wks spon lab	193/6466	194/6473	32.5	3.0	1.0
Group 4a: Multip (excl prev caesarean sections) single ceph ≥ 37 wks induced	49/1297	49/1298	6.5	3.8	0.2
Group 4b: Multip (excl prev caesarean sections) single ceph ≥ 37 wks CS before lab	260/260	261/261	1.3	100.0	1.3
Group 5: Previous caesarean section single ceph ≥ 37 wks	987/1281	991/1285	6.4	77.1	5.0
Group 6: All nulliparous, single breeches	447/499	449/501	2.5	89.6	2.3
Group 7: All multiparous, single breeches (incl previous caesarean sections)	222/266	223/267	1.3	83.5	1.1
Group 8: All multiple births (incl previous caesarean sections)	239/393	240/394	2.0	60.9	1.2
Group 9: All single abnormal lies (incl previous caesarean sections)	26/26	26/26	0.1	100.0	0.1
Group 10: All single ceph ≤ 36 wks (incl previous caesarean sections)	265/947	266/949	4.8	28.0	1.3
Not stated	17/32	-	-	-	-
**Total**			**100.0**		**20.5**

Note: missing data are imputed using the observed distributions for caesarean births, then for non-caesarean births.

Table S17. Robson's Ten Group Classification System, including sub-groups, in Sweden 2015

	Number of CS/ all women in each group	Number of CS/ all women in each group imputed data	Relative size of each group (%)	CS rate in each group (%)	Contribution of each group (%)
Group 1: Nullip single ceph ≥ 37 wks spon lab	2935/34958	2936/34968	30.4	8.4	2.6
Group 2a: Nullip single ceph ≥ 37 wks induced	2149/8591	2150/8594	7.5	25.0	1.9
Group 2b: Nullip single ceph ≥ 37 wks CS before lab	1380/1380	1380/1380	1.2	100.0	1.2
Group 3: Multip (excl prev caesarean sections) single ceph ≥ 37 wks spon lab	755/41091	755/41102	35.7	1.8	0.7
Group 4a: Multip (excl prev caesarean sections) single ceph ≥ 37 wks induced	356/7256	356/7258	6.3	4.9	0.3
Group 4b: Multip (excl prev caesarean sections) single ceph ≥ 37 wks CS before lab	1385/1385	1385/1385	1.2	100.0	1.2
Group 5: Previous caesarean section single ceph ≥ 37 wks	5518/10348	5520/10351	9.0	53.3	4.8
Group 6: All nulliparous, single breeches	1880/2018	1881/2019	1.8	93.2	1.6
Group 7: All multiparous, single breeches (incl previous caesarean sections)	1066/1250	1066/1250	1.1	85.3	0.9
Group 8: All multiple births (incl previous caesarean sections)	918/1669	918/1669	1.5	55.0	0.8
Group 9: All single abnormal lies (incl previous caesarean sections)	257/303	257/303	0.3	84.8	0.2
Group 10: All single ceph ≤ 36 wks (incl previous caesarean sections)	1442/4717	1443/4719	4.1	30.6	1.3
Not stated	7/33	-	-	-	-
**Total**			**100.0**		**17.4**

Note: missing data are imputed using the observed distributions for caesarean births, then for non-caesarean births.

Table S18. Robson's Ten Group Classification System, including sub-groups, in Switzerland 2014

	Number of CS/ all women in each group	Number of CS/ all women in each group imputed data	Relative size of each group (%)	CS rate in each group (%)	Contribution of each group (%)
Group 1: Nullip single ceph ≥ 37 wks spon lab	3206/20311	3400/21523	25.7	15.8	4.1
Group 2a: Nullip single ceph ≥ 37 wks induced	2413/8513	2559/9022	10.8	28.4	3.1
Group 2b: Nullip single ceph ≥ 37 wks CS before lab	2573/2573	2729/2729	3.3	100.0	3.3
Group 3: Multip (excl prev caesarean sections) single ceph ≥ 37 wks spon lab	845/19956	896/21144	25.3	4.2	1.1
Group 4a: Multip (excl prev caesarean sections) single ceph ≥ 37 wks induced	315/5746	334/6088	7.3	5.5	0.4
Group 4b: Multip (excl prev caesarean sections) single ceph ≥ 37 wks CS before lab	2362/2362	2505/2505	3.0	100.0	3.0
Group 5: Previous caesarean section single ceph ≥ 37 wks	5887/6831	6244/7244	8.7	86.2	7.5
Group 6: All nulliparous, single breeches	2553/2628	2708/2787	3.3	97.2	3.2
Group 7: All multiparous, single breeches (incl previous caesarean sections)	1169/1254	1240/1330	1.6	93.2	1.5
Group 8: All multiple births (incl previous caesarean sections)	1250/1550	1326/1644	2.0	80.7	1.6
Group 9: All single abnormal lies (incl previous caesarean sections)	2530/3562	2683/3776	4.5	71.1	3.2
Group 10: All single ceph ≤ 36 wks (incl previous caesarean sections)	1560/3644	1655/3863	4.6	42.8	2.0
Not stated	135/4727		-	-	-
**Total**			**100.0**		**33.8**

Note: missing data are imputed using the observed distributions for caesarean births, then for non-caesarean births. Because of the low proportion of missing data for caesareans, we first imputed the missing data into caesarean/non-caesarean using the overall caesarean rate.
